# Management of intraocular pressure elevation during hemodialysis of neovascular glaucoma: a case report

**DOI:** 10.1186/s12886-016-0199-z

**Published:** 2016-03-05

**Authors:** P. Frezzotti, C. Menicacci, S. A. Bagaglia, P. Mittica, F. Toto, I. Motolese

**Affiliations:** Ophthalmology Unit, Department of Medicine, Surgery and Neuroscience, University of Siena, Viale Bracci s.n.c., 53100 Siena, Italy

**Keywords:** Neovascolar glaucoma, Hemodialysis, Intra ocular pressure

## Abstract

**Background:**

It is generally accepted that dialysis may lower plasma osmolality at a faster rate than changes in ocular osmolality. This osmotic difference causes water to migrate from the plasma into the aqueous humor, increasing intraocular pressure. Certain authors have described IOP increase in patients with narrow angles.

**Case presentation:**

Here we report a neovascular glaucoma patient who experienced a substantial increase in IOP associated with severe eye pain and blurred vision during sessions of dialysis. The patient had been refractory to several antiglaucoma drugs and improved after intravenous administration of 20 % hyperosmotic glucose solution with dialysis and pan-retinal photocoagulation.

**Conclusion:**

It is the first report in which intravenous glucose administration and reduction of neovascularization by argon laser pan-retinal photocoagulation successfully managed IOP increase during dialysis in neovascular glaucoma. Further clinical studies are required to confirm our results.

## Background

Eye complications may occur during or after hemodialysis in patients with end stage renal disease [1]. Ocular hypertension has been reported in subjects without glaucoma [2–5] and is more accentuated in patients with glaucoma [6, 7]. Reports of intraocular pressure (IOP) rise during dialysis in neovascular glaucoma are extremely limited [8]. It is generally accepted that dialysis may lower plasma osmolality at a faster rate than changes in ocular osmolality. This osmotic difference causes water to migrate from the plasma into the aqueous humor, increasing intraocular pressure [9]. This theory has not however been clearly demonstrated and some studies have indicated that IOP increase is minimal or non existent, probably due to improvement in dialysis techniques. [10] Certain authors have described IOP increase in patients with narrow angles [11, 12]. Here we report a neovascular glaucoma patient who experienced a substantial increase in IOP associated with severe eye pain and blurred vision during sessions of dialysis. The patient had been refractory to several antiglaucoma drugs and improved after intravenous administration of 20 % hyperosmotic glucose solution with dialysis and pan-retinal photocoagulation. To our knowledge this is the first report in which intravenous glucose administration and reduction of neovascularization by argon laser pan-retinal photocoagulation successfully managed neovoscalar glaucoma.

## Case presentation

**Fig. 1:**
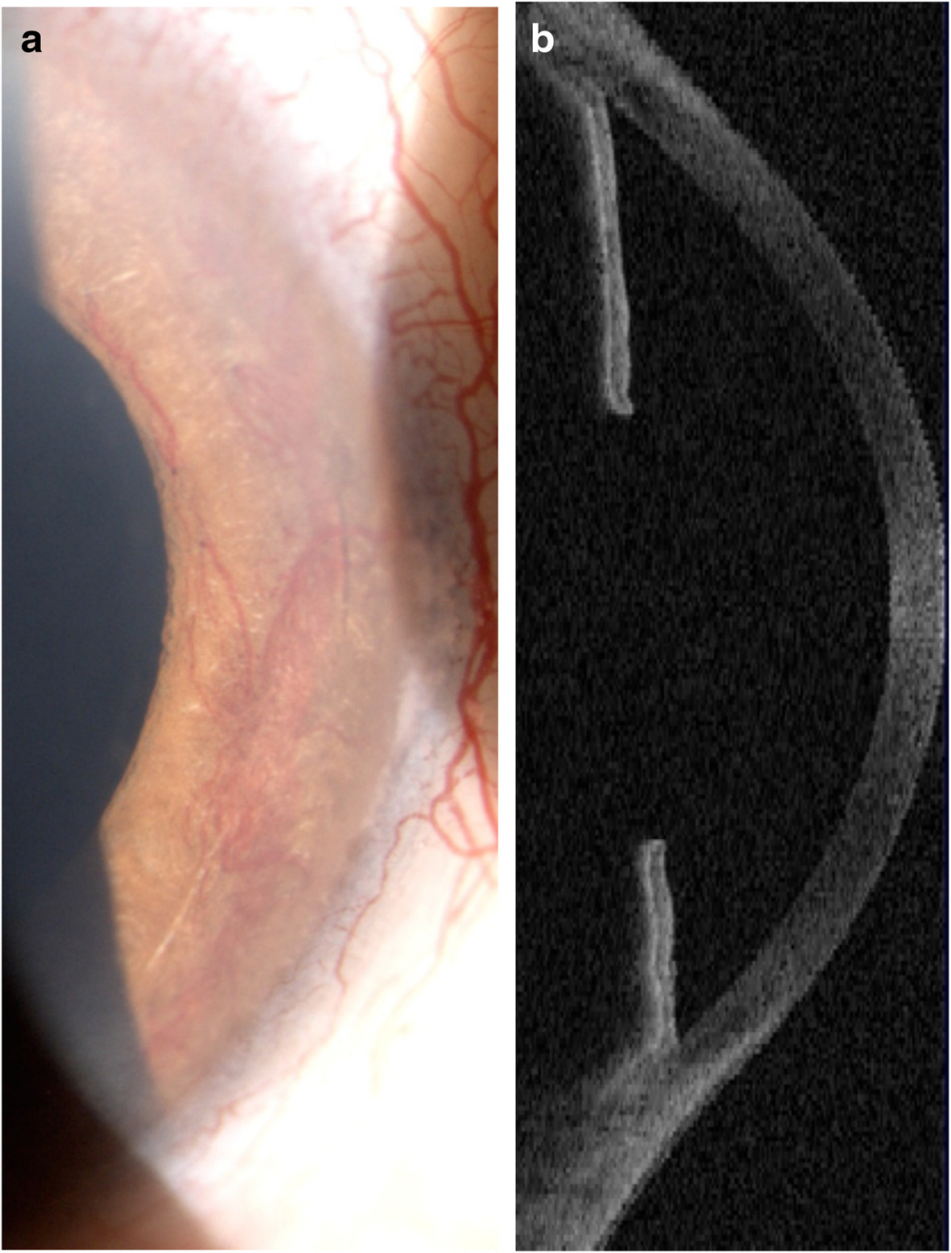
Biomicroscopic (**a**) and OCT (**b**) images of anterior chamber showing angle closure due to neovascularization of the angle

**Fig. 2:**
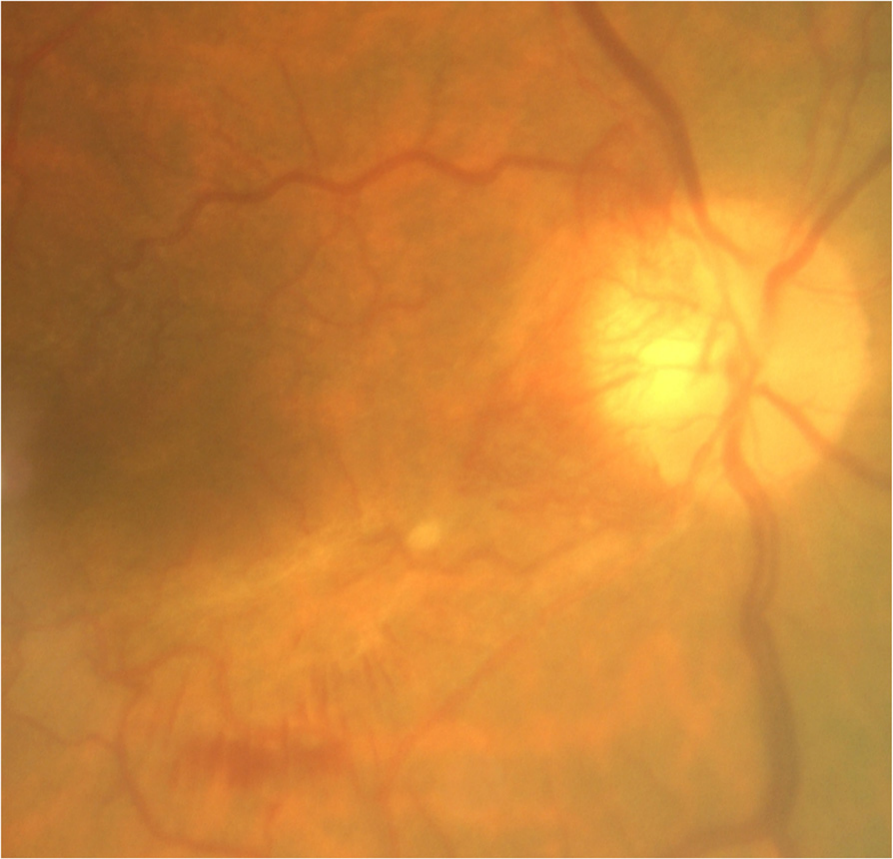
Appearance of ONH in right eye

The patient was a 48 year old male with end-stage renal disease secondary to single pelvic Kidney, hypertension and hypertriglyceridemia. Medical history included renal failure in 2007 after kidney transplant, regular hemodialysis in 1989 and home dialysis five times a week since 2014. On 10 0ctober 2014, he was referred to our glaucoma unit because of severe ocular pain and blurred vision during a session of dialysis. The blurred vision resolved spontaneously within 2 h of onset. Nine years prior to this presentation he had undergone cataract removal in both eyes with intraocular lens implant in the right eye (RE) and aphakia in the left eye (LE). He was first diagnosed with neovascular glaucoma in 2013. Combined topical ß-blocker and carbonic anhydrase inhibitor (Cosopt®) and prostaglandin analogue (Travatan®) were prescribed for both eyes when intraocular pressure reached 38 and 34 mmHg in the right and left eyes, respectively. On presentation, ophthalmological examination showed 2/20 best correct visual acuity (BCVA) in the right and blindness in the left eye. Anterior segment examination showed iris rubeosis in both eyes (Fig. 1a): gonioscopy revealed neovascularization spreading completely over the iridocorneal angle with peripheral anterior synechiae at trabecular level on the nasal and temporal sides, and OCT showed evidence of “false angle” (Fig. 1b). Intraocular pressure measurements were 22 mmHg RE and 21 mmHg LE. When alpha_2_-agonist (Alphagan®) and pilocarpine hydrochloride 2 % eye drops were applied to both eyes, intraocular pressure decreased to 16 mmHg RE and 15 mmHg LE, but blurred vision recurred in the right eye during dialysis. The 30-2 Humphrey visual field test showed a large arcuate scotoma in the upper and lower hemifields. Perimetric defect type and stage were graded as mixed 2 by the Brusini Glaucoma Staging System 2 [13]. In the left eye it was impossible to perform visual field examination because of poor visual acuity. Fundoscopic examination revealed ischemic retinopathy with pre-retinal new vessels in the optic disk area of both eyes (Fig. 2). An epiretinal membrane covered the macular area of the right eye and ischemic peripheral retinal areas were observed. Seven years earlier the patient suffered occlusion of the central retinal vein in the left eye, followed in January 2014 by ischaemic optic neuropathy. We began argon laser treatment of ischemic peripheral retinal areas.

**Fig. 3:**
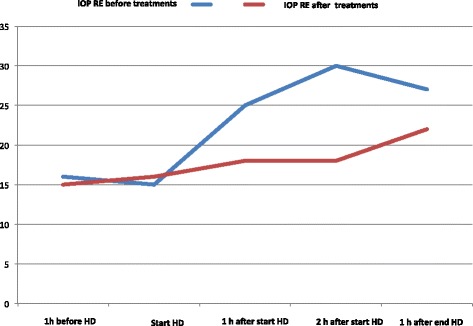
Differences between IOP before and after i.v. glucose and panretinal photocoagulation treatments

Intraocular pressure was measured five times during hemodialysis for a 1-week period: at the beginning of dialysis, at hourly intervals during dialysis, at the end, and 1 and 2 h after the end of dialysis. Average intraocular pressure in the right eye at the beginning, after 2 h of dialysis, at the end and 2 h later was 15, 30, 27 and 22 mmHg, respectively. Thus intraocular pressure in the right eye increased by 15 mmHg (100 %) during hemodialysis. In the left eye it remained normal. Topical instillation of eye drops did not prevent IOP increase in the right eye. Hemodialysis was performed routinely five times a week for 2 h and 40 min using central venous catheter for chronic pyelonephritis using NxStage System One (NSO) artificial kidney. After failure of eye treatment, we administered intravenous glucose (20 % glucose at 100 mL/h) during dialysis to prevent IOP rise. We perform argon laser treatment between 60 and 30 days before dialysis. Eye pain and blurred vision resolved. We repeated IOP measurement during dialysis as above and recorded mean values of 15, 16 and 18 mmHg at the beginning, 2 h later and at the end of dialysis, respectively. Differences between IOP before and after i.v. glucose and panretinal photocoagulation treatments are shown in Fig. 3. In the last ophthalmological evaluation on 17 February 2015, BCVA was 2/20 in RE, anterior segment examination showed reduction of iris rubeosis in RE, IOP was 8 mmHg RE and 9 mmHg LE and fundoscopic examination revealed successful argon laser treatment of ischaemic peripheral retinal areas in both eyes. The optic disk appear unchanged and the visual field stable after the two treatments. Alpha_2_-agonist (Alphagan®) and pilocarpine hydrochloride 2 % eye drops were discontinued. IOP was 13 mmHg RE and 12 mmHg LE. No pain or blurred vision occurred again.

### Discussion

Intraocular pressure may increase during hemodialysis, as reported in many patients with and without glaucoma. Many mechanisms have been invoked to explain this phenomenon [1–12], the most frequent being that IOP increases due to a rapid fall in plasma osmolality that stimulates increased formation of aqueous humor [2, 3, 9, 13]. This relationship between plasma osmolality and IOP during hemodialysis has been studied since the report of Sitprija et al. [14, 15]. Some authors have suggested that decreased aqueous outflow might be the mechanism of intradialytic IOP increase, since most patients who showed IOP elevation during dialysis also had a narrow anterior chamber angle [16, 17]. Rever et al. [18] reported that anterior chamber depth decreased significantly during dialysis. In the present neovascular glaucoma patient, we suggest that raised IOP resulted from an imbalance between aqueous outflow, obstructed by angle closure due to new blood vessels, and production of aqueous humor. The rise in IOP during dialysis sustains this explanation: the drainage system was unable to compensate the increase in aqueous production during dialysis. Argon laser retinal photocoagulation is known to reduce angle neovascularization induced by peripheral retinal ischemia in neovascular glaucoma patients [19, 20].

Several therapeutic solutions have been recommended to prevent a symptomatic increase in IOP during dialysis sessions. Medical expedients include topical and systemic carbonic anhydrase inhibitor, which is relatively contraindicated in dialysis patients as it can precipitate severe metabolic acidosis, which might be fatal to patients with end-stage renal disease [11]. Seo et al. [21] used an oral hypertonic solution with glycerol that proved effective for IOP control and safer in glaucoma patients. Other therapeutic approaches were described by Jaeger et al. who administered intravenous mannitol, which reduced IOP but predisposed patients to various side effects [17]. Other solutions include medical therapy with topical Beta blockers [5], argon laser trabeculoplasty for exfoliative glaucoma [6], Ahmed valve implant for neovascular glaucoma [8] and trabeculectomy for diabetic retinopathy [22]. In our case, elevated IOP levels during hemodialysis were refractory to topical treatments and the patient’s young age and neovascular glaucoma are both well-known risk factors for bleb fibrosis and surgical failure [23, 24].

Intravenous glucose administration was recently proposed to manage IOP increase during dialysis [25]. The authors showed that administration of 20 % glucose solution (100 mL/h) at each dialysis session prevented increased production of aqueous humor due to relative serum hypo-osmolality. After this change of treatment IOP remained in the normal range. One limitation of high glucose administration could be the overall increase in serum glucose in diabetic patients. The most important side effect to be examined, in patients with chronic kidney failure, is that chronic use of hyperosmotic solutions does not reduce the effectiveness of hemodialysis. Effectiveness of hemodialysis is calculated by artificial kidney’s software and it depends on three parameters: BUN values before and after hemodialysis sessions; body weight; time of hemodialysis sessions. In our experience effectiveness of hemodialysis has not changes until now and in literature there aren’t studies on chronic use of hyperosmotic solutions in these type of patients. Another important side effect to be examined is the possible establishment of hyperglycemia but also in this case literature has not provided us more information due to poor series but our patient has not had alterations on glycemia values or on glucose intolerance and performs bi-weekly all tests blood needed.

Before attempting surgery we decided to administer intravenous glucose and to perform argon laser treatment of ischemic peripheral retinal areas to reduce neovascularization of the occluded angle. The synergy of the two treatments, one aimed at reducing the production of aqueous humor and the other at improving its outflow, proved effective in controlling IOP during the dialysis session and was safe for our neovascular glaucoma patient with end-stage renal disease.

## Conclusions

Our case of recurrent painful IOP spikes during dialysis in a patient with neovascular glaucoma unresponsive to conventional medical treatment is the first report in which intravenous glucose administration and reduction of neovascularization by argon laser pan-retinal photocoagulation successfully managed IOP increase during dialysis. Further clinical studies are required to confirm our results.

## Consent

The patient has given a written consent for the publication of this case report.
